# Congenital fibrovascular pupillary membrane with secondary glaucoma and associated fundus anomalies: a case report and literature review

**DOI:** 10.1186/s12886-026-05193-x

**Published:** 2026-08-03

**Authors:** Yanyan Cheng, Ying Yang, Lihui Yin, Mingyu Ren, Juan Yin, Congying Wang, Yi Gao

**Affiliations:** 1 Hebei Eye Hospital, No. 399 Quanbei East Street, Xingtai, Hebei Province 054000 China; 2 Department of Ophthalmology, Hebei Medical University, No. 361 Zhongshan East Road, Chang’an District, Shijiazhuang, Hebei Province 050017 China; 3 Tianjin Eye Hospital, No. 4 Gansu Road, Heping District, Tianjin, Tianjin, 300020 China

**Keywords:** Congenital fibrovascular pupillary membrane, Secondary glaucoma, Case report

## Abstract

**Background:**

Congenital fibrovascular pupillary membrane (CFPM) is a rare unilateral congenital anterior segment dysplasia, mainly characterized by the presence of fibrous vascular membrane-like tissue in the pupil area. This membrane can obstruct the pupil, impede aqueous humor circulation, and progress to severe secondary glaucoma. This report describes a rare case of CFPM-induced secondary glaucoma coexisting with fundus anomalies.

**Case presentation:**

A 5-month-old female infant presented with right eye photophobia and epiphora, which had developed 20 days prior. Ophthalmological examination revealed the following: right eye intraocular pressure (IOP) 27.4 mmHg, corneal opacity and enlargement, near-complete absence of the anterior chamber, occluded pupillary membrane, and other intraocular structures were not visible. The left pupil was mildly irregular in shape, but the remaining ocular structures were unremarkable. B-scan ultrasonography demonstrated a deep, localized excavation of the posterior globe in the right eye. The patient was diagnosed with secondary glaucoma (right eye) and congenital fibrovascular pupillary membrane (right eye). Surgical intervention was performed under general anesthesia, comprising pupillary membrane resection, pupiloplasty, trabeculotomy and trabeculectomy in the right eye. Histopathological examination of the excised tissue revealed fibrovascular proliferation, consistent with congenital vascular fibrous pupillary membrane. Genetic testing revealed no pathogenic variants. At the 17-month postoperative follow-up, the right eye had an IOP of 10 mmHg without no membrane recurrence. Fundus photography of the right eye revealed a pale optic disc with an enlarged cup-to-disc ratio, indistinct retinal vessels, a large excavation involving the optic disc and surrounding tissues with patchy pigmentation within them, and two focal white lesions in the inferior retina that appeared scleral.

**Conclusion:**

We report a clinically rare case of CFPM-induced secondary glaucoma, with concurrent fundus anomalies. Comprehensive ophthalmic evaluation is essential for CFPM patients, with attention to the possibility of posterior segment involvement. For severe cases, timely surgical intervention is recommended to preserve intraocular anatomical structures, with emphasis on long-term postoperative follow-up and the management of complications.

**Supplementary Information:**

The online version contains supplementary material available at https://doi.org/10.1186/s12886-026-05193-x.

## Background

Congenital fibrovascular pupillary membrane (CFPM) is characterized by persistent embryonic fibrovascular tissue in the pupillary region, extending from the iris to the anterior lens capsule, with adhesions to the lens capsule. It may be associated with goniodysgenesis [1]. Typically unilateral and sporadic, CFPM is progressive, obstructing the pupil and impairing vision [2]. It is a rare but high-risk cause of secondary glaucoma. Particularly in infants, if not managed promptly, it can lead to irreversible optic nerve damage and vision loss.

The nomenclature has evolved through several terms including: “Congenital pupil-iris-lens membrane with goniodysgenesis” [3], “Congenital idiopathic microcoria” [4], and “Fibrovascular congenital iris membrane with pupillary distortion” [5]. The currently accepted terminology is CFPM. The current literature primarily consists of case reports and small-sample studies; clinical epidemiological data are sparse, and the incidence rate remains unclear.

Pathogenetically, Cibis et al. proposed defective differentiation, migration, and regression of neural crest-derived tissue as the underlying mechanism [3]. However, Lambert’s histopathological analysis demonstrated negative neuronal-specific enolase staining (a neural crest marker) and morphological similarities to persistent fetal vasculature (PFV), suggesting this may represent a distinct PFV subtype [6]. In 2022, Zhao Peiquan’s research team introduced the term anterior-anterior persistent fetal vasculature (AAPFV) to classify this entity as a PFV variant characterized by more anterior involvement of the iris and lens anterior capsule compared to conventional anterior PFV [7].

CFPM is primarily characterized as an isolated anomaly of the anterior segment, involving the iris, pupil, and anterior lens capsule. The prevailing literature supports that it infrequently co-occurs with posterior segment abnormalities [8]. We report a rare case of a 5-month-old female infant with CFPM that progressively developed into pupillary seclusion, anterior chamber collapse, and secondary glaucoma, accompanied by fundus anomalies.

## Case presentation

A 5-month-old female infant presented with right eye photophobia and epiphora, which had developed 20 days prior. The patient had a history of miosis and inferior pupil displacement in her right eye at birth which was found by her parents but wasn’t given treatment.

The infant was born at full term via spontaneous vaginal delivery, with no history of trauma. Her mother had no history of fever and no exposure to radiation or chemical drugs during pregnancy. The parents were non-consanguineous. There was no family history of ocular diseases or genetic disorders in the parents and other family members.

**Fig. 1:**
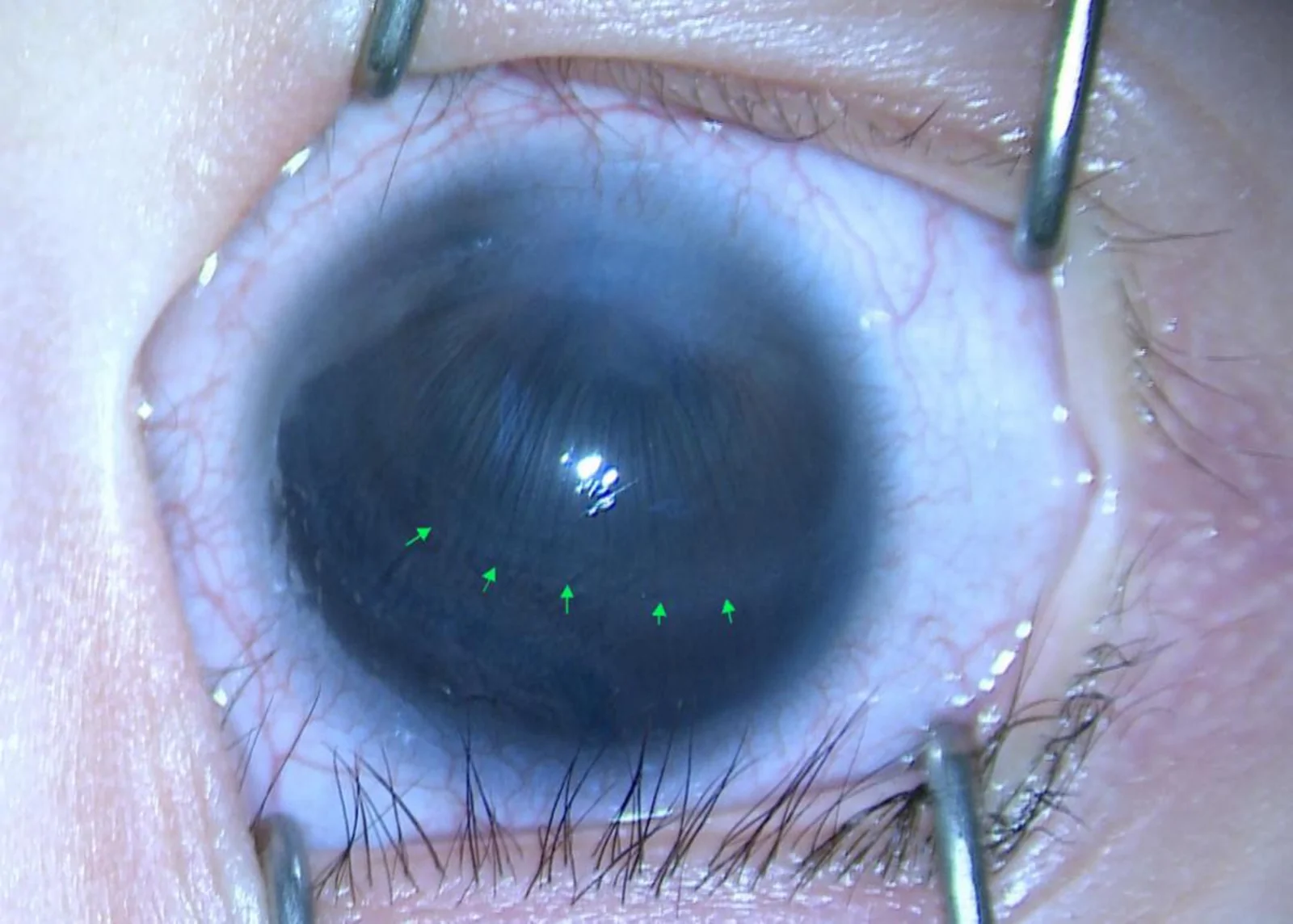
Right eye: near-complete absence of the anterior chamber and Haab’s striae (green arrow)

**Fig. 2:**
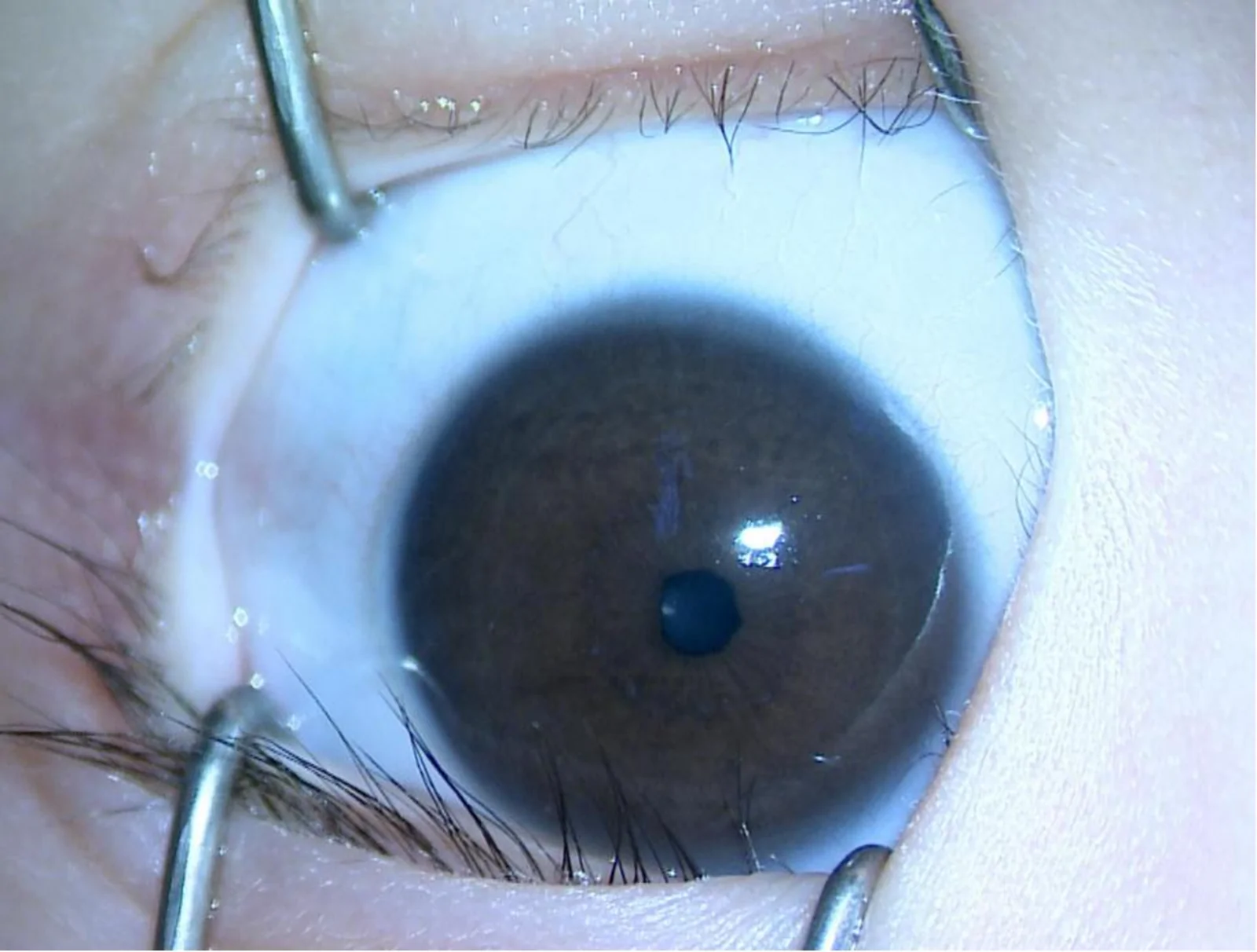
Left eye: mildly irregular pupil

**Fig. 3:**
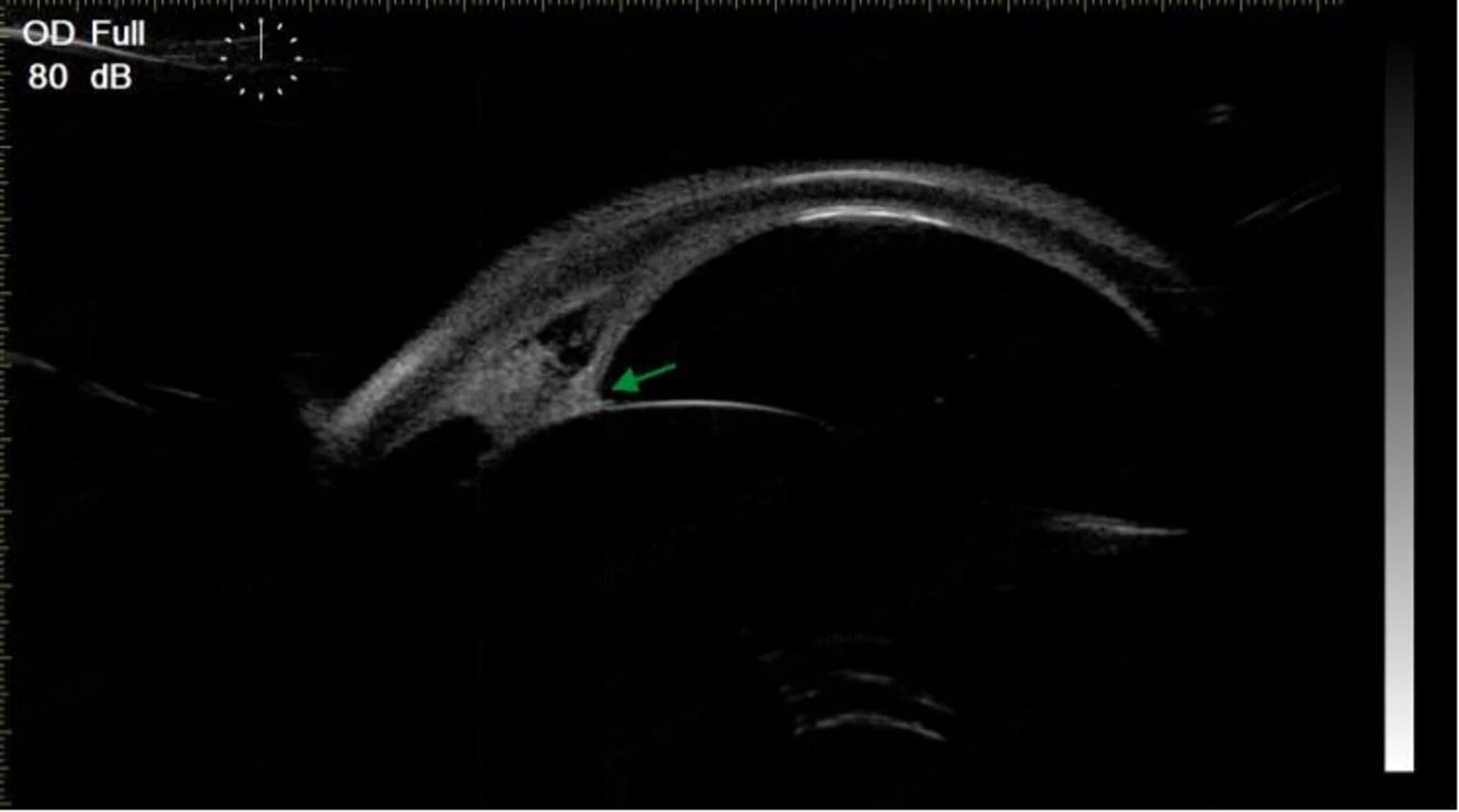
UBM (right eye): near-complete absence of the anterior chamber, iris thinning, a membranous structure posterior to the inferior iris, lens adherent to both the iris and membranous structure (green arrow)

**Fig. 4:**
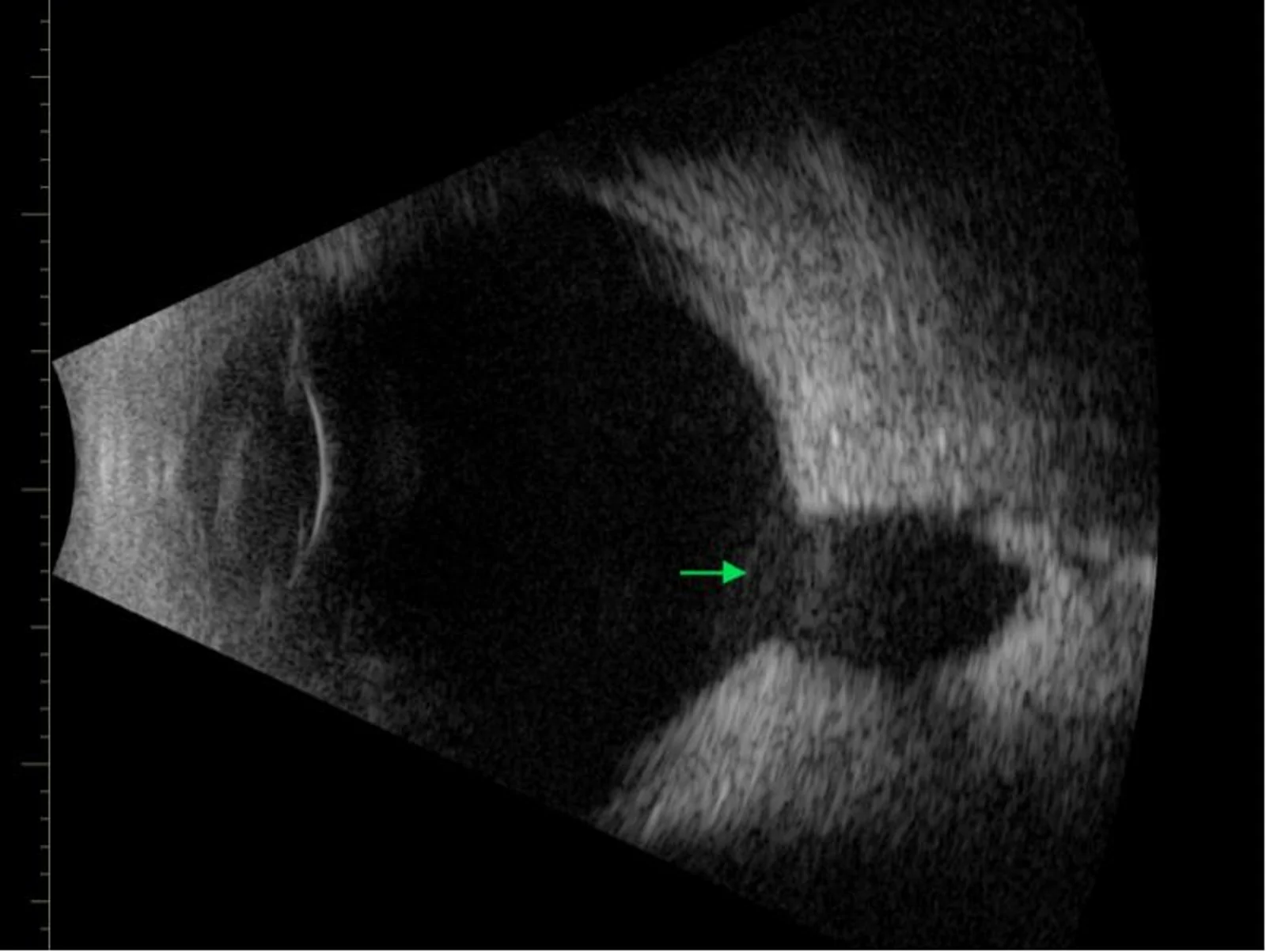
B-scan ultrasonography (right eye): a deep, localized excavation of the posterior globe (green arrow)

A comprehensive ophthalmologic examination was performed. The infant showed no light response in the right eye, while the left eye was able to track objects. Intraocular pressure (IOP) measurements obtained with iCare tonometry demonstrated significant asymmetry: 27.4 mmHg in the right eye versus 14.6 mmHg in the left eye while the child was in a state of natural sleep and without anesthesia. The right eye exhibited corneal opacity, corneal enlargement, Haab’s striae, and a broadened limbus. There was a near-complete absence of the anterior chamber in the right eye, with inferior iris displacement and stromal thinning, precluding detailed posterior segment evaluation (Fig. 1). In contrast, the left eye demonstrated transparent cornea, mildly irregular pupil, normal anterior chamber depth and clear lens (Fig. 2). Fundus examination of the left eye demonstrated a normal retinal appearance. Gonioscopy revealed open angles in the left eye. Ultrasound biomicroscopy (UBM) of the right eye demonstrated: near-complete absence of the anterior chamber, iris thinning, a membranous structure posterior to the inferior iris, lens adherent to both the iris and membranous structure (Fig. 3). A-scan ultrasonography revealed axial length asymmetry: 23.70 mm in the right eye versus 20.76 mm in the left eye. B-scan ultrasonography demonstrated a deep, localized excavation of the posterior globe in the right eye, measuring 8.52 mm × 4.71 mm (Fig. 4). Subsequent genetic testing revealed no pathogenic variants.

**Fig. 5:**
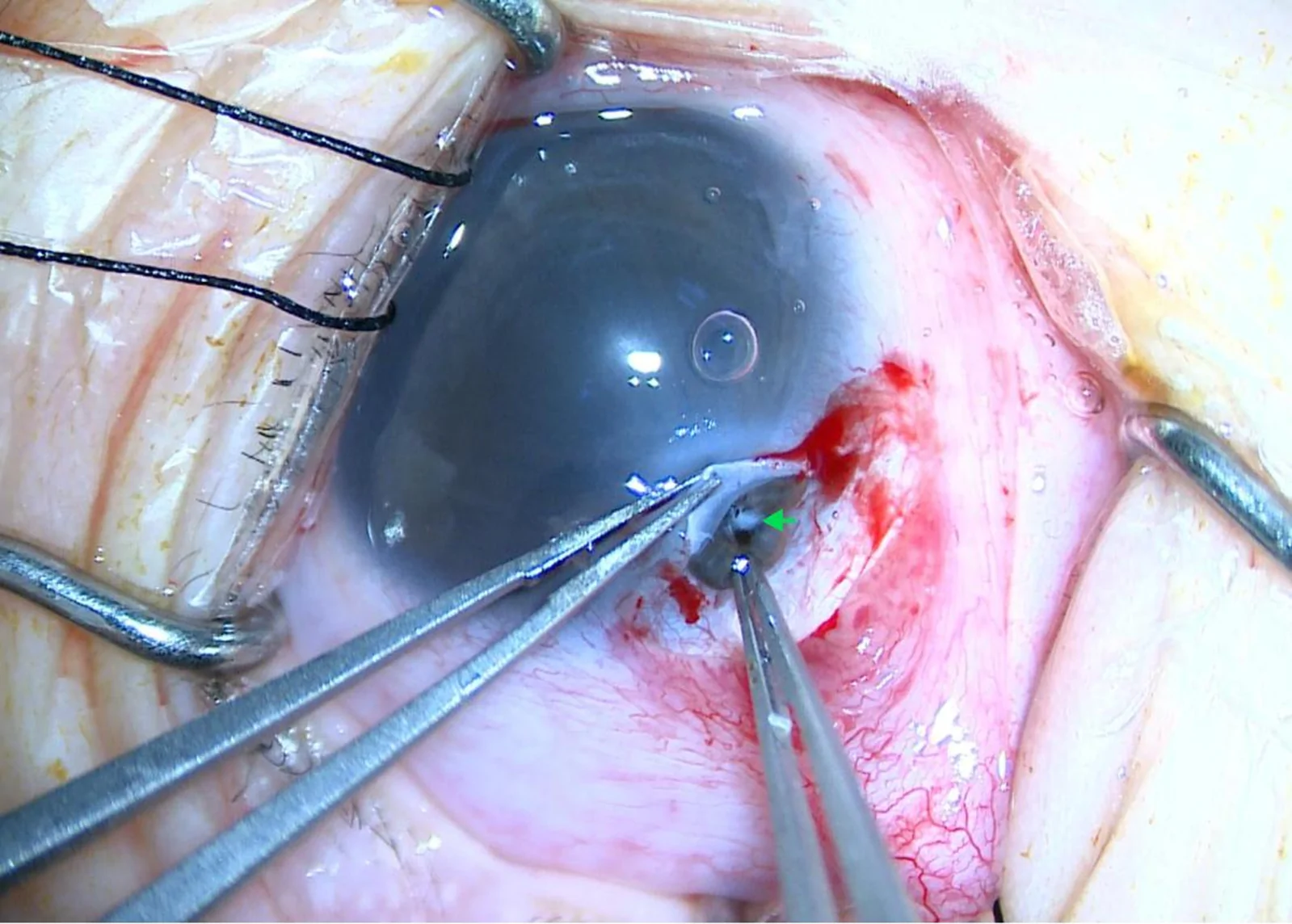
Right eye: white membrane in the pupillary area (green arrow)

**Fig. 6:**
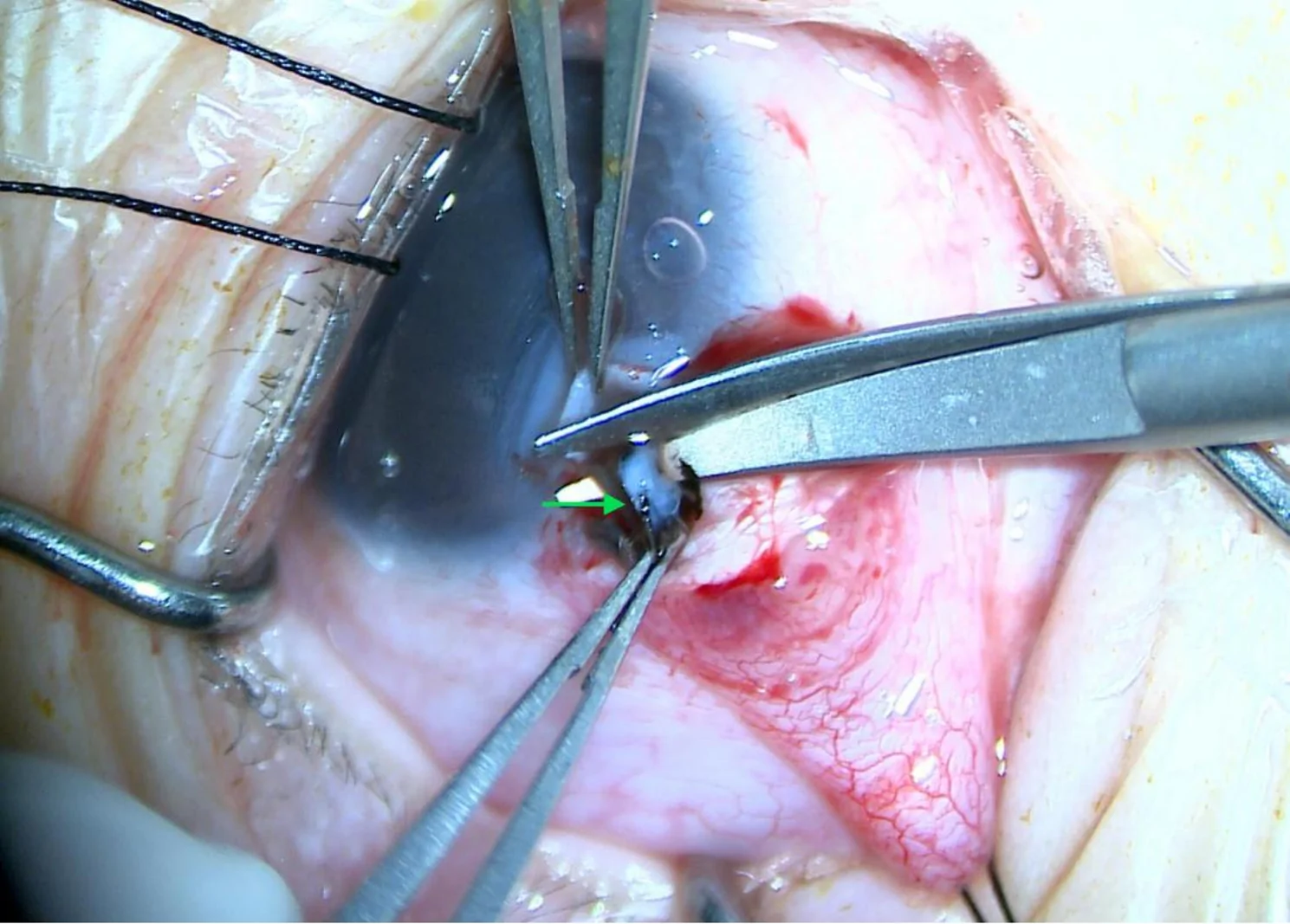
Right eye: completely excised membranous structure (green arrow)

**Fig. 7:**
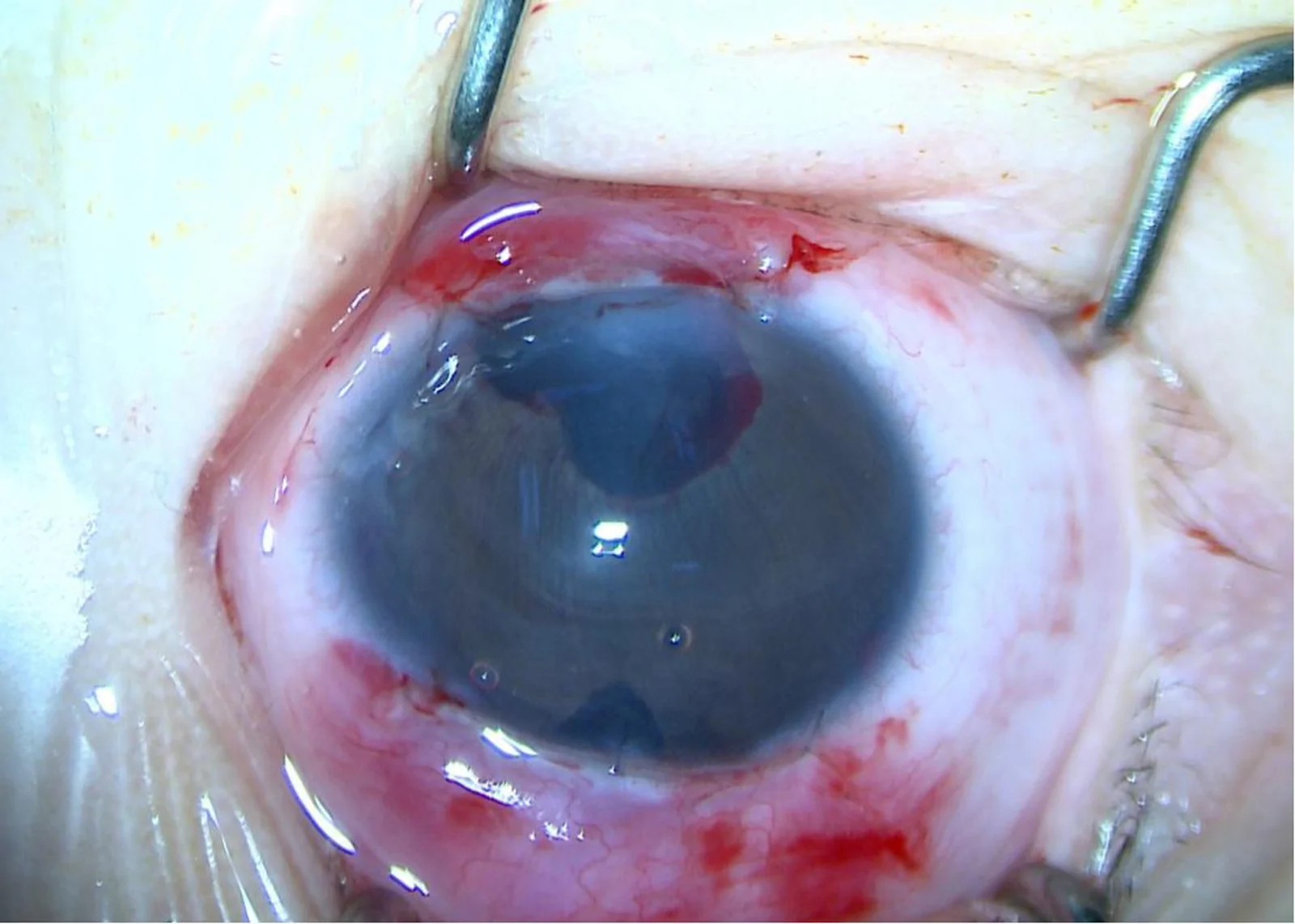
Right eye: formed anterior chamber and pupil

**Fig. 8:**
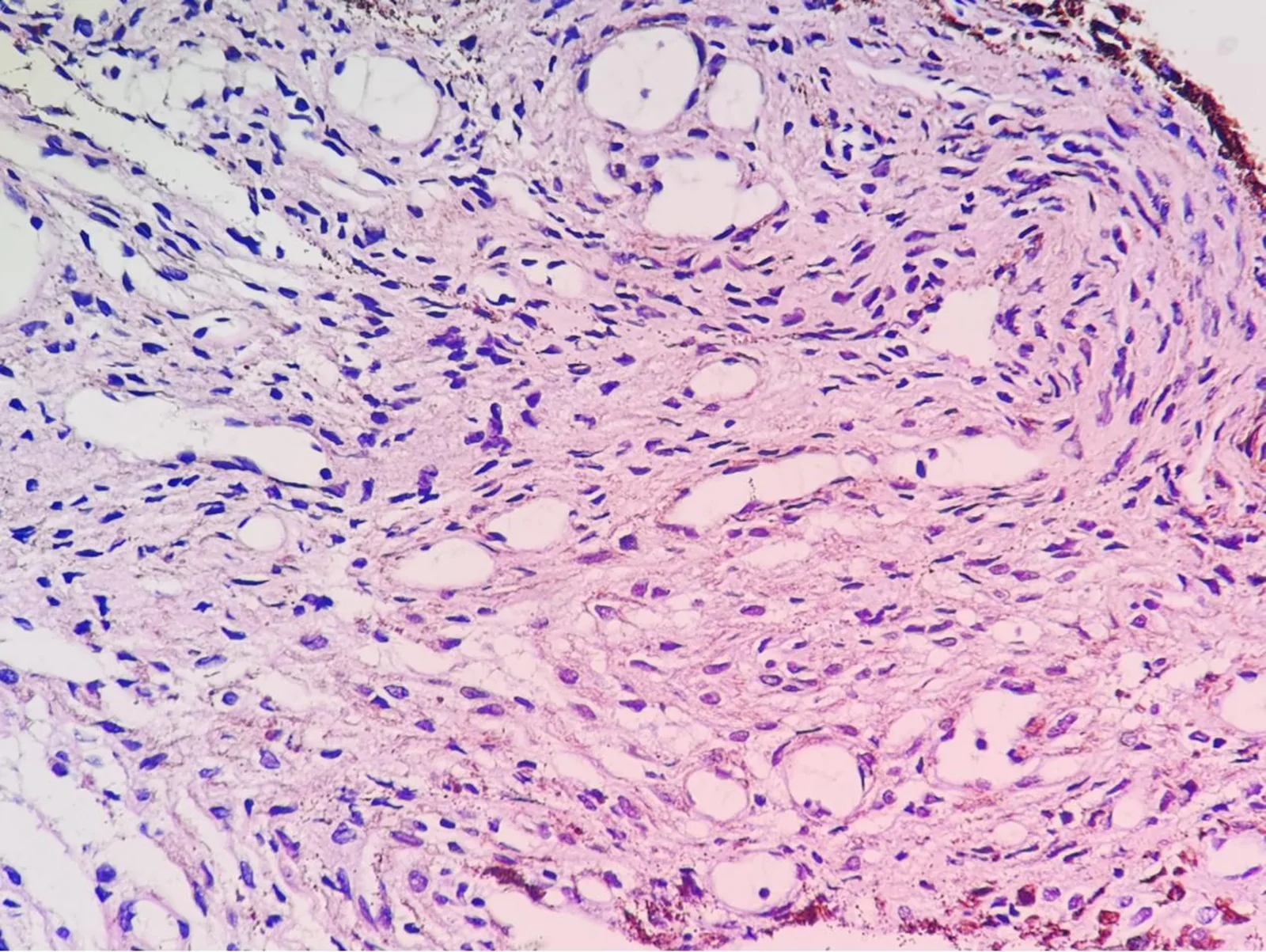
Histopathological examination: fibrovascular proliferation with focal pigment deposition(×400)

**Fig. 9:**
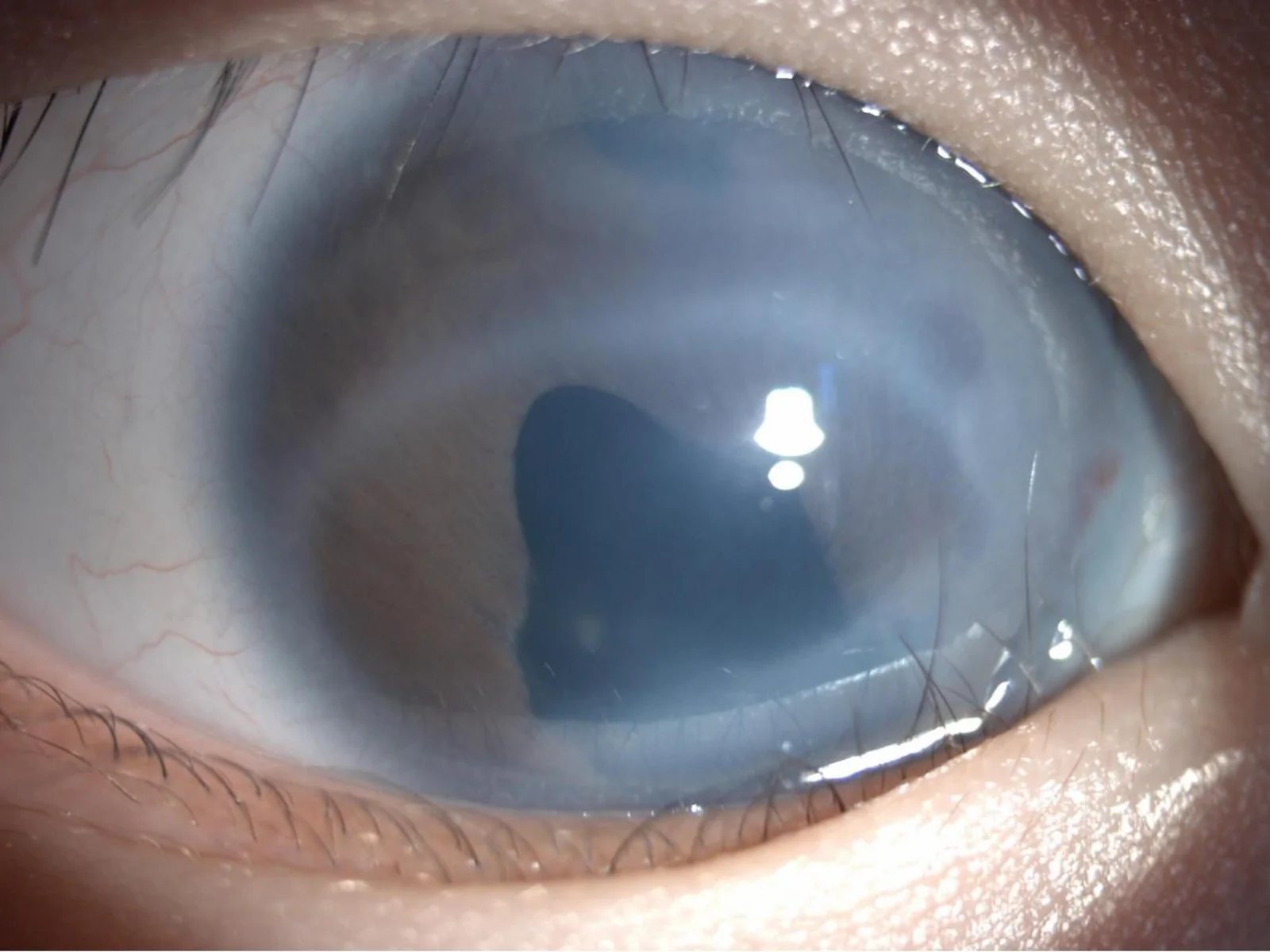
Right eye: formed but irregular pupil at 1-month postoperative follow-up

**Fig. 10:**
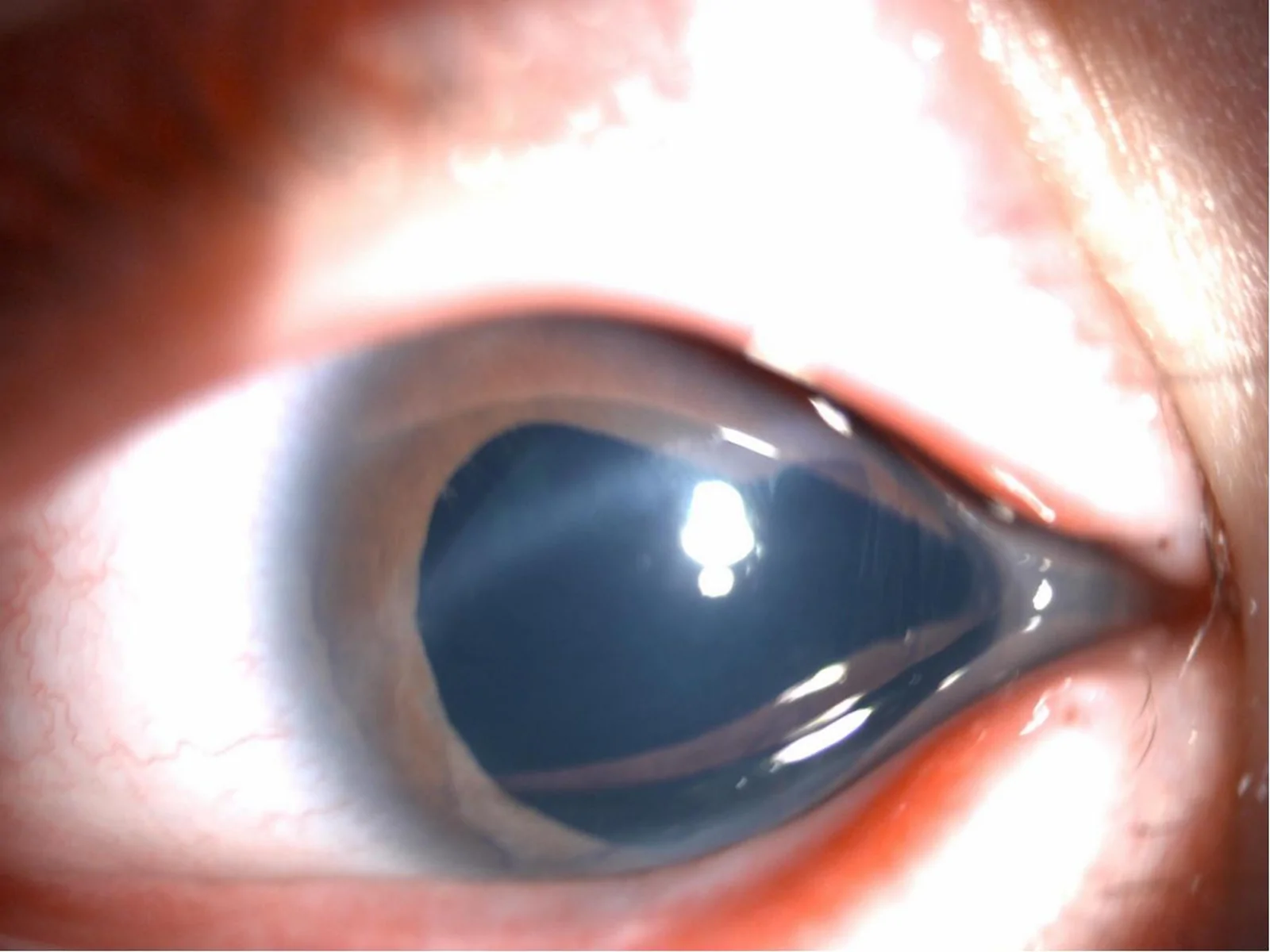
Right eye: well-formed anterior chamber, clear lens, no membrane recurrence, and improved corneal clarity at the 17-month postoperative follow-up

**Fig. 11:**
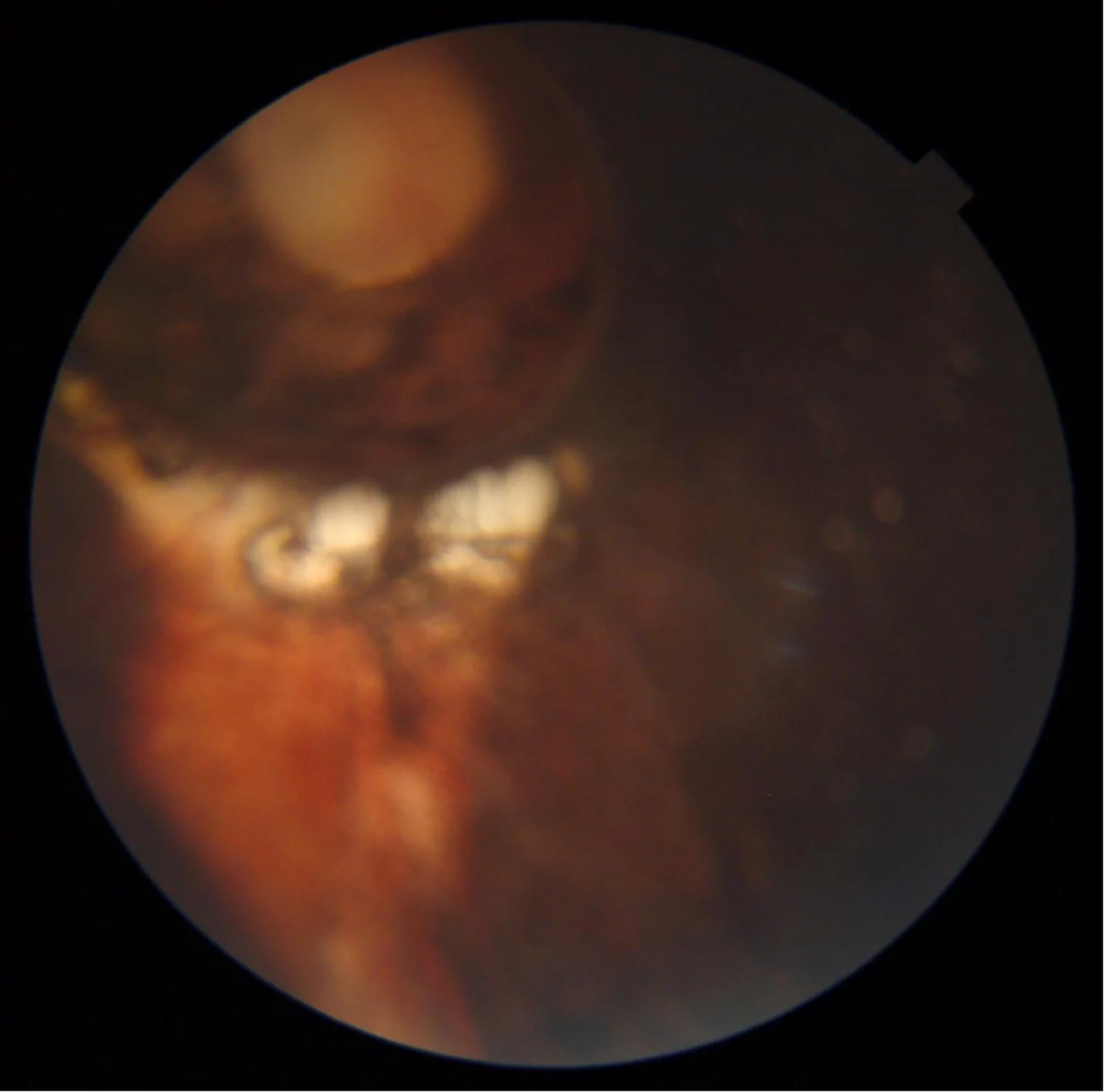
Right eye: a pale optic disc with an enlarged cup-to-disc ratio, indistinct retinal vessels, a large excavation involving the optic disc and surrounding tissues with patchy pigmentation within them, and two focal white lesions in the inferior retina that appeared scleral

Based on ophthalmic examination, the admission diagnoses were CFPM and secondary glaucoma in the right eye. Under general anesthesia, the patient underwent pupillary membrane resection, pupiloplasty, trabeculotomy, and trabeculectomy in the right eye. No antimetabolites were used during the surgical procedure. Intraoperatively, a white membrane was identified in the pupillary area at the 6 o’clock position through the inferior incision (Fig. 5), extending into the posterior chamber. The adhesion between this membranous structure and the lens was loose and not firm. The membrane was completely and smoothly excised (Fig. 6). A trabeculotomy combined with trabeculectomy was then performed at the 12 o’clock position through the superior incision. At the conclusion of the surgery, both the anterior chamber and the pupil were formed (Fig. 7). Histopathological examination of the excised pupillary membrane tissue revealed fibrovascular proliferation with focal pigment deposition (Fig. 8), consistent with the clinical diagnosis of CFPM. At the 1-month postoperative follow-up, IOP was 14.9 mmHg in the right eye without the need for any anti-glaucoma medications and 13.8 mmHg in the left eye. Anterior segment photography of the right eye showed a formed but irregular pupil and a well-formed anterior chamber (Fig. 9). At the 17-month postoperative follow-up, the right eye had an IOP of 10 mmHg without medications, a well-formed anterior chamber, a clear lens, no membrane recurrence, and improved corneal clarity (Fig. 10). B-scan ultrasonography showed no appreciable changes in the posterior segment. Fundus photography of the right eye revealed a pale optic disc with an enlarged cup-to-disc ratio, indistinct retinal vessels, a large excavation involving the optic disc and surrounding tissues with patchy pigmentation within them, and two focal white lesions in the inferior retina that appeared scleral (Fig. 11). There were no postoperative surgical complications.

### Discussion and conclusion

This report describes an infant with CFPM-induced secondary glaucoma, presenting with a near-complete absence of the anterior chamber, inferior iris displacement, and fundus anomalies. The case successfully underwent combined surgical intervention with regular follow-up. It is clinically notable for illustrating complex pediatric ocular abnormalities; the surgical management and outcomes offer a valuable reference for treating similar cases.

There have been rare reports of CFPM coexisting with optic nerve hypoplasia in the literature [9]. We conducted a thorough literature review and identified a total of 132 confirmed cases of CFPM (Table 1). Of these, 31 cases (23.5%) exhibited documented fundus abnormalities. Among these 31 cases, 27 presented with glaucomatous optic neuropathy (e.g., pale optic disc and glaucomatous cupping), which was attributed to elevated IOP secondary to glaucoma. The remaining 4 cases were suspected to have congenital anomalies of the optic nerve, such as optic nerve hypoplasia and morning glory syndrome, although the original authors did not provide detailed explanations for these findings. In our case, fundus photography of the right eye showed a pale optic disc and an enlarged cup-to-disc ratio, suggestive of glaucomatous damage attributable to elevated IOP. Nevertheless, fundus examination revealed an extremely large and deep excavation involving the optic disc and surrounding tissues. B-scan ultrasonography measured the excavation at 8.52 mm × 4.71 mm, dimensions far exceeding the physiological range of optic disc cupping. The excavation could be due to posterior staphyloma or coloboma. Moreover, two focal white lesions with a scleral appearance were observed in the inferior retina, which are likely scleral show, a finding commonly seen in pathological myopia. Concurrently, elevated IOP may further deepen the glaucomatous cupping and exacerbate retinal nerve fiber layer damage, particularly in the presence of pre-existing structural vulnerability.

**Table 1:** Summary of previously reported CFPM cases and associated fundus abnormalities

First author(et al.), Year	Number of Cases	Cases with Fundus Abnormalities	Specific Findings
Cibis et al. 1986 [1]	2	0	
Liang et al. 2021 [2]	33	4	Pale optic disc
Cibis et al. 2004 [3]	9	0	
Lambert et al. 1988 [4]	5	0	
Robb et al. 2001 [5]	7	0	
Lambert et al. 2012 [6]	6	0	
Ren et al. 2022 [7]	11	1	Morning glory syndrome
Demidenko et al. 2009 [9]	1	1	Optic nerve hypoplasia
Zhu et al. 2021 [10]	32	22	Glaucomatous cupping
Al-Otaibi et al. 2023 [11]	2	1	Optic disc cupping around 0.8
Zhou et al. 2022 [12]	3	0	
Deshpande et al. 2006 [13]	1	0	
Wang et al. 2020 [14]	4	0	
Ramappa et al. 2012 [15]	1	0	
Shao et al. 2025 [16]	13	2	Retinal pigment disorders and the peripheral retina and optic disk dysplasia
Oltra et al. 2015 [17]	1	0	
Kesarwani et al. 2009 [18]	1	0	
Total	132	31	

Given the broad differential diagnosis of congenital ocular anomalies, we considered Axenfeld-Rieger syndrome, Peters anomaly, PFV, and persistent pupillary membrane (PPM). In our patient, the absence of posterior embryotoxon, systemic findings, and positive family history effectively excluded Axenfeld-Rieger syndrome; the lack of central corneal opacification and prominent iridocorneal synechiae argued against Peters anomaly; the classic triad of PFV—retrolental fibrovascular membrane, posterior cataract, and persistent hyaloid artery—was not observed; and the simple iris-to-iris strands typical of PPM were absent. To provide a transparent framework for this differential diagnosis, the key distinguishing features of each entity are summarized in Table 2. Despite excluding these classic entities, the concurrent anterior segment dysplasia and fundus anomalies suggest that our patient’s condition may represent a broader spectrum of iridocorneal dysgenesis, warranting further investigation.

**Table 2:** Differential diagnosis between the present case and other congenital ocular anomalies

Disorder	Primary Lesion	Laterality	Lens Status	Glaucoma	Systemic Associations
Present case	Fibrovascular pupillary membrane	Unilateral	Clear	Yes	None
Peters anomaly	Central corneal opacity with Descemet membrane defect	Type I: unilateral Type II: bilateral	Type I: Clear Type II: cataract /corneolenticular touch	Possible	Possible
Axenfeld-Rieger syndrome	Posterior embryotoxon and iris hypoplasia	Bilateral	Usually clear	Possible	Yes
PFV	Retrolental fibrovascular membrane	Usually unilateral	Cataract (common)	Possible	None
PPM	Vascularized iris strands across the pupil	Usually bilateral	Usually clear	Rare	None

Zhu Yingting et al. developed a classification system for CFPM-induced secondary glaucoma based on iris configuration and anterior chamber depth (UBM findings), categorizing cases into three distinct types: type I (“U” shape), type II (“Y” shape), and type III (no anterior chamber), respectively accounted for 53%, 34% and 13% [10]. Progression from type I to type III demonstrates worsening disease severity, characterized by increased iris bombé and progressive shallowing of central anterior chamber depth. According to the report by Zhu Yingting [19], All 25 patients with CFPM-induced secondary glaucoma underwent anterior segment reconstruction (ASR). Among these cases, type Ⅰ eyes showed the highest success rate at 72.7%, compared with 18.2% for type Ⅱ and 0% for type Ⅲ. Most type Ⅱ eyes and all type Ⅲ eyes required additional glaucoma surgery. CFPM-induced secondary glaucoma is associated with pupillary block and angle closure. However, not all patients achieve IOP normalization after relief of pupillary block. Goniodysgenesis may also contribute to elevated IOP. In our case, the patient underwent UBM examination, which revealed near-complete obliteration of the anterior chamber in the right eye, consistent with type Ⅲ. Considering the universally poor surgical outcomes of ASR in type Ⅲ cases, the potential coexistence of goniodysgenesis in CFPM, and the risk of repeated anesthesia exposure associated with staged procedures, we opted to perform combined membrane excision and glaucoma surgery instead of a staged approach. The combined approach simultaneously addressed the key issues: relieving pupil block and iris traction (via membrane excision and pupiloplasty) and establishing an aqueous outflow pathway (via glaucoma surgery), which optimized the conditions for visual system preservation. Intraoperatively, several critical issues required careful management. Precise excision of the pupillary membrane was difficult due to the absence of the anterior chamber and adhesions between the membrane and the lens, requiring meticulous dissection and timely replenishment of viscoelastic in the anterior chamber to avoid iatrogenic injury to the lens and corneal endothelium. Performing angle surgery in an enlarged infant eye with anatomical distortion complicated the accurate localization of the trabecular meshwork.

In children with CFPM, the lens typically remains clear; therefore, combined lens surgery is generally unnecessary [11, 12]. However, form deprivation from congenital pupillary obstruction puts these patients at risk of amblyopia, and early recognition and surgical management of dense membranes obscuring the pupillary axis is crucial [13]. The assessment of visual prognosis in this pediatric patient is limited owing to poor cooperation and insufficient objective visual function testing. Coexisting fundus anomalies further increase the uncertainty of visual outcomes. Individualized amblyopia management is critical for maximizing visual function and optimizing long-term visual prognosis. Close collaboration among ophthalmologists and parents is essential. Serial examinations should be supplemented stepwise during long-term follow-up, with timely adjustment of treatment strategies to achieve the best possible visual outcomes.

In summary, CFPM is a rare yet potentially blinding progressive anterior segment disorder that can lead to secondary glaucoma and may be accompanied by fundus anomalies. Comprehensive ophthalmic evaluation is essential for CFPM patients, with attention to the possibility of posterior segment involvement. For severe cases, timely surgical intervention is recommended to preserve intraocular anatomical structures. Postoperative care requires establishment of a close monitoring protocol focusing on anatomical structural changes, IOP control, and vigilance for potential complications including membrane recurrence, strabismus, and amblyopia.

## Supplementary Information

Below is the link to the electronic supplementary material.

Supplementary Material 1

## Data Availability

The data supporting the findings of this study are available from the corresponding author upon reasonable request.

## Abbreviations

AAPFV: Anterior-anterior persistent fetal vasculature
ASR: Anterior segment reconstruction
CFPM: Congenital fibrovascular pupillary membrane
IOP: Intraocular pressure
PFV: Persistent fetal vasculature
PPM: Persistent pupillary membrane
UBM: Ultrasound biomicroscopy
